# *Haemophilus ducreyi* Bacteria Causing Cutaneous Ulcer Outbreak in Children, Sierra Leone, December 2025

**DOI:** 10.3201/eid3210.260727

**Published:** 2026-10

**Authors:** Jianxu Zhang, Peng Lv, Teng Zhao, Hanji Jiang, James S. Squire, Martin S. Yonnie, Zikan Koroma, Ibrahim F. Kamara, Abdulai A. Jalloh, Guangqian Pei, Yunfei Wang, Hongling Liu, Mohamed B. Jalloh, Zhiqiang Mi

**Affiliations:** Changchun Veterinary Research Institute, Chinese Academy of Agricultural Sciences, Changchun, China (J. Zhang); National Key Laboratory of Advanced Biotechnology, Academy of Military Medical Sciences, Beijing, China (P. Lv); State Key Laboratory of Pathogen and Biosecurity, Academy of Military Medical Sciences, Beijing (T. Zhao, H. Jiang, G. Pei, Y. Wang, Z. Mi); University of Sierra Leone, Freetown, Sierra Leone (J.S. Squire); National Public Health Agency, Freetown (J.S. Squire, Z. Koroma, A.A. Jalloh, M.B. Jalloh); Ministry of Health, Bombali DHMT, Makeni, Sierra Leone (M.S. Yonnie); World Health Organization, Health Systems and Services Cluster, Freetown (I.F. Kamara); The Fifth Medical Center of Chinese PLA General Hospital, Beijing (H. Liu); College of Medicine and Allied Health Sciences, University of Sierra Leone, Freetown (M.B. Jalloh)

**Keywords:** Haemophilus ducreyi, bacteria, cutaneous ulcer, outbreak, children, Sierra Leone

## Abstract

We report a cutaneous ulcer outbreak in Sierra Leone in 2025 that predominantly affected ≈403 schoolchildren. The causative agent was confirmed as *Haemophilus ducreyi*, a bacterium traditionally associated with the sexually transmitted infection chancroid but more recently recognized as an emerging cause of lower limb cutaneous ulcers in tropical regions.

Nongenital cutaneous ulcers as a public health problem commonly affect children in many tropical regions, especially in West and Central Africa, the South Pacific, and South Asia (*1*). Yaws, which is caused by *Treponema pallidum* subspecies *pertenue*, has historically been considered the primary causative agent of such ulcers (*2*). In recent years, however, several studies have demonstrated that *Haemophilus ducreyi* is a leading bacterial cause of cutaneous ulcers (lower leg lesions in the absence of genital lesions) in yaws-endemic regions (*3*–*5*). 

Beginning on October 29, 2025, several clustered cases of lower limb cutaneous ulcers predominantly affecting schoolchildren in the Kayassie community, Safroko Limba Chiefdom, Bombali District, Sierra Leone, were reported to the National Public Health Agency of Sierra Leone. The infections were originally suspected to be *Bacillus anthracis* infection. Restriction of close student-to-student contact was advised for school management, while immediate laboratory testing for pathogen identification was launched. The outbreak showed sustained transmission: 266 cases by December 24, 2025; 386 cases by January 13, 2026; and 403 cases by February 10, 2026. Patients showed progressive skin ulcerations, sometimes associated with localized edema. The ulcers predominantly occurred on the lower limbs, where opportunistic skin injuries could easily develop, and commonly manifested as a localized lesion with an irregular border. The base of the ulcer was mostly composed of dry and crusted tissue without a purulent exudate (Figure 1). Recovery progressed steadily after case treatment and management (Appendix). Skin swab sample collection was conducted with consent from all participants or their parents or guardians, and the study was conducted using deidentified data derived from routine public health programmatic activities and interventions. The study was also reviewed and approved administratively by the National Public Health Agency of Sierra Leone.

**Figure 1:**
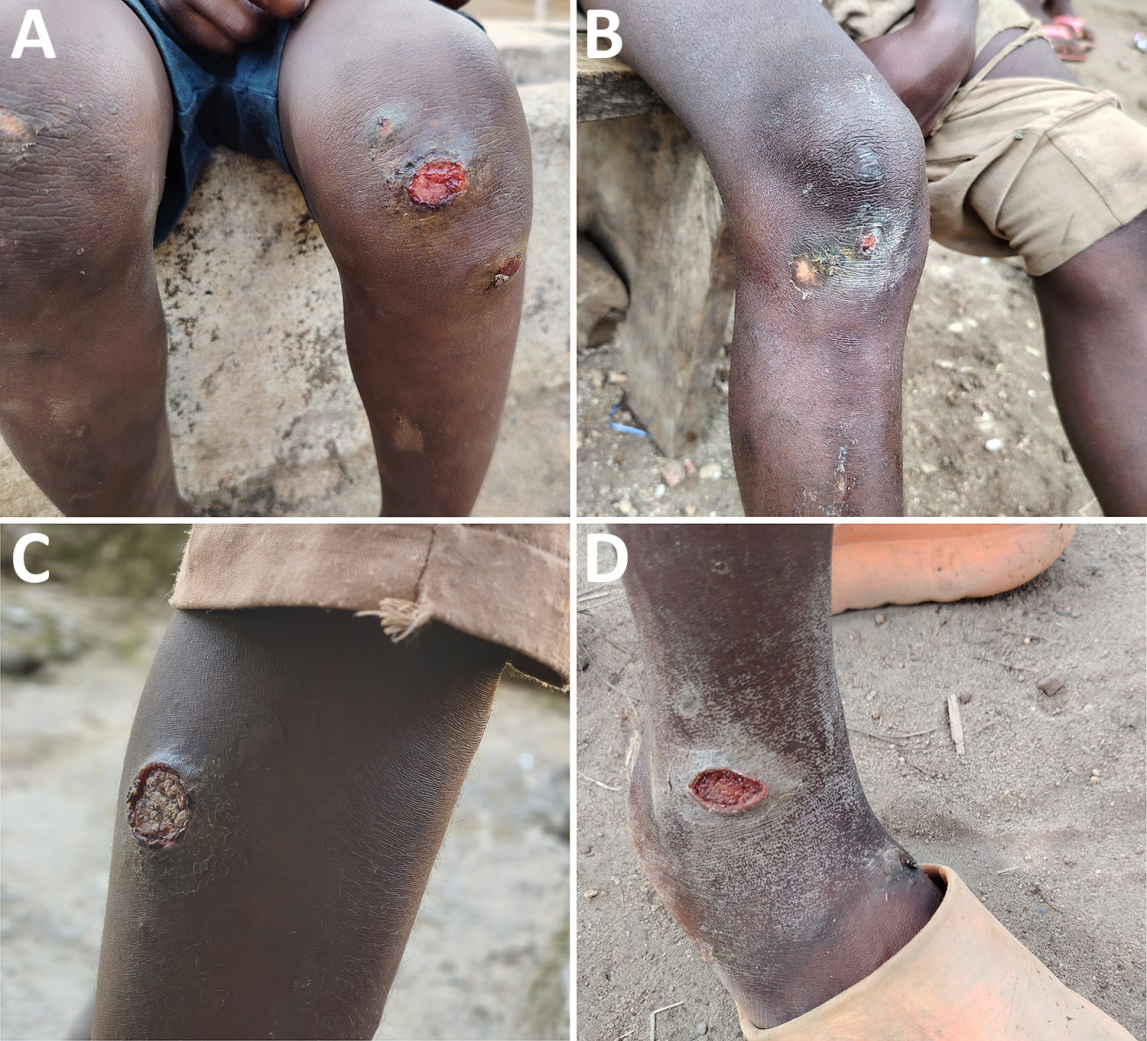
Irregular appearance of cutaneous ulcer lesions from patients in study of *Haemophilus ducreyi* bacteria causing cutaneous ulcer outbreak in children, Sierra Leone, December 2025. A) Lesions over the knee. B) Lesions below the knee. C) Lesions on the mid-lateral aspect of the leg. D) Lesions at the ankle.

*B. anthracis* infection was immediately excluded in the first 7 skin swab samples by using quantitative PCR targeting *rpoB*, *pagA*(pXO1), and *capC*(pXO2) (BioPerfectus Technologies, https://www.biosearchtech.com). Analysis of metagenomic sequence data produced from MinION (Oxford Nanopore Technologies, https://nanoporetech.com) (Appendix) revealed that *H. ducreyi* sequences were present in 4 of the 7 swab samples; no other relevant pathogens were identified (Appendix Figure 1). To confirm sequencing results, we performed real-time PCR according to a previous report (*6*). All of the first 7 samples tested positive for *H. ducreyi. *Those preliminary results demonstrated that *H. ducreyi* was highly associated with this cutaneous ulcer outbreak. 

As the outbreak progressed, another 149 swab samples were collected and shipped to the laboratory for PCR testing. Of the 156 total samples, 96 (61.5%) tested positive for *H. ducreyi*. No DNA evidence of *T. pallidum* subsp. *pertenue*, subsp. *pallidum*, or subsp. *endemicum* bacteria was detected by quantitative PCR in any of the 156 samples (*7*). 

To further determine the etiologic agent causing this outbreak, we sequenced genomic DNA from 22 swab samples (12 *H. ducreyi*–positive and 10 *H. ducreyi*–negative) on the DNBSEQ-G99 platform (MGI Tech, https://www.mgi-tech.com) (Appendix). Metagenomic analysis indicated no evidence of *T. pallidum* subsp. *pertenue* in the 22 samples, which is consistent with the PCR result. We identified no other etiologic pathogen associated with cutaneous ulcers in the 22 samples. The most abundant bacterial species were *Staphylococcus aureus* and *Klebsiella pneumoniae* (Figure 2). 

**Figure 2:**
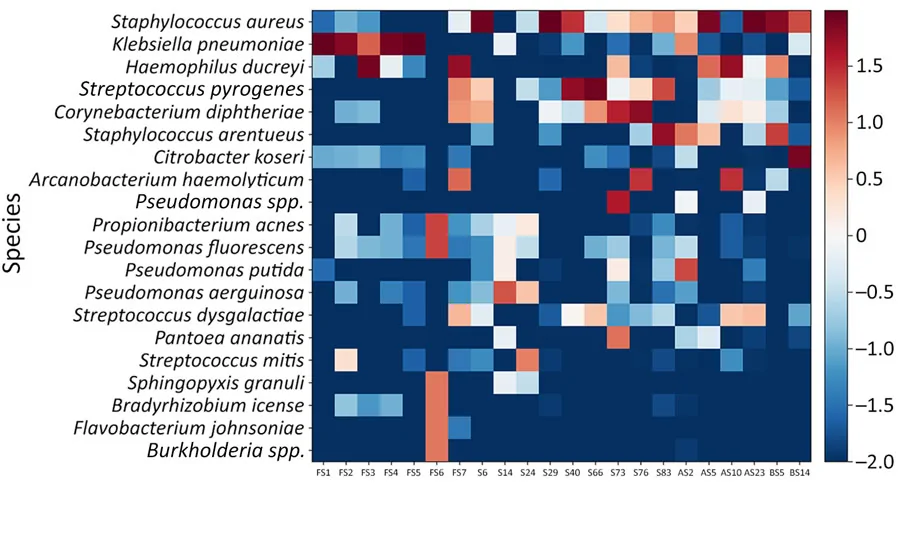
Heatmap of bacterial species investigated from study of *Haemophilus ducreyi* bacteria causing cutaneous ulcer outbreak in children, Sierra Leone, December 2025. Values are shown as log_10_(abundance + 0.01)%. Metagenomic analysis based on the DNBSEQ-G99 sequencing platform (MGI Tech, https://www.mgi-tech.com). The heatmap revealed that *H. ducreyi*, the third most abundant bacterium, was the most suspected causative agent through 22 skin swab samples. The samples were designated as follows: FS, samples collected on November 18, 2025; S, samples collected on December 12, 2025; AS and BS, samples collected on December 18, 2025.

To isolate *H. ducreyi*, we immediately inoculated 61 swab samples in the field onto Columbia blood agar base (Thermo Fisher Scientific, https://www.thermofisher.com), supplemented with 2.5% fresh yeast extract, 3 µg/mL of vancomycin, and 30% rabbit blood (*8*). After 96 hours of microaerophilic culture at 33°C, grayish-white pinpoint-sized colonies were picked up (*9*). After 3 consecutive subcultures, we confirmed PCR-positive colonies to be *H. ducreyi* (Appendix Figure 2). Among the 61 inoculated samples, *H. ducreyi* grew from 9 samples. Ambient transport temperature and lack of CO_2_ might have reduced isolation. 

We sequenced the complete genomes of 9 *H. ducreyi* strains on an Illumina platform (https://www.illumina.com) (Appendix). Phylogenetic analysis demonstrated that the 9 isolates recovered in Sierra Leone formed a cluster with strains from Ghana and were distributed into 2 separate clades (Appendix Figure 3). We submitted short reads for 22 skin swab samples and 9 strains of *H. ducreyi* bacteria to the National Center for Biotechnology Information Sequence Read Archive (https://www.ncbi.nlm.nih.gov/sra; accession no. PRJNA1450830). 

To try to determine a source for the outbreak, we tested 60 saline-soaked fly samples, 8 water samples from neighboring ponds, and tissue samples from 2 goats that died during the outbreak by quantitative PCR. No samples were positive for *H. ducreyi*.

This investigation confirmed that *H. ducreyi* was the causative bacterial agent of the cutaneous ulcer outbreak among children in Bombali District, Sierra Leone, in 2025. Sierra Leone is historically classified as a yaws-endemic country (*10*), and national eradication campaigns have been conducted in past decades. This outbreak, in essence, was a protracted, low-severity outbreak of cutaneous *H. ducreyi* infection, yielding no fatalities and leading to gradual resolution under enhanced surveillance and case management. However, the source of *H. ducreyi* in this outbreak remains unclear. Clinicians should be aware of the possibility of cutaneous *H. ducreyi* infection in yaws-endemic regions.

Appendix Additional information on *Haemophilus ducreyi* bacteria causing cutaneous ulcer outbreak in children, Sierra Leone, December 2025.
