# Supplementary material for: Haemophilus ducreyi Bacteria Causing Cutaneous Ulcer Outbreak in Children, Sierra Leone, December 2025
Source: Emerg Infect Dis. 2026 Oct;32(10):1764–7. doi: 10.3201/eid3210.260727 (PMC13632863; doi:10.3201/eid3210.260727)
Supplement: Supplementary file 1 [file 26-0727-Techapp-s1.pdf]

Article DOI: https://doi.org/10.3201/eid3210.260727

EID cannot ensure accessibility for supplementary materials supplied by authors. Readers who have difficulty accessing supplementary content should contact the authors for assistance.

Haemophilus ducreyi Bacteria Causing Cutaneous Ulcer Outbreak in Children, Sierra Leone, December 2025

Appendix

Case treatment and management

Prior to laboratory confirmation of *H. ducreyi*, all patients received standardized wound care and symptomatic management. This included daily wound dressing with povidone-iodine for participants residing within a 2-km radius of the health facility, and three times per week for those living beyond 2 km. Symptomatic treatment comprised a single dose of loratadine (5 mg for children <5 years, 10 mg for those ≥5 years) for itching, paracetamol as needed for pain/fever, vitamin C 50 mg daily for 1–2 weeks, and routine multivitamins for patients with pallor. Following molecular confirmation of *H. ducreyi*, azithromycin was added at 30 mg/kg bodyweight in a single oral dose for mild ulcers, or in a 3-day course for severe ulcers. Recovery progressed steadily-368 patients had fully recovered by 10 February 2026, leaving ≈35 active cases. The outbreak originated in Safroko Limba Chiefdom, Bombali District, and later spread to Mara Chiefdom in Bombali District and to neighboring Tonkollili District. Children aged 5–15 years are the most affected group, with males accounting for 56% of all cases.

Nanopore sequencing

A nanopore sequencing library was prepared by using the DNA samples and the Rapid Barcoding and Sequencing kits provided by Oxford Nanopore Technologies (Oxford, UK). The library was sequenced on a MinION Mk1B device with an R9.4.1 flow cell. The acquisition of raw data, base calling, and demultiplexing were performed in real time with MinKNOW 24.11.8 software. The passed bases of 2.76 Mb were generated from 7 swab samples at the default minimum Q score of 8. The obtained reads were then classified with the software Centrifuge (release 1.0.3) against the "Bacteria, Archaea, Viruses, Human (compressed)" database (Available from: http://ccb.jhu.edu/software/centrifuge/).

Metagenomic sequencing

Metagenomic DNA extracted from samples was subjected to high-throughput sequencing on the DNBSEQ-G99 platform. Standard paired-end sequencing was performed for 300 cycles. Raw signal data were processed through the built-in BasecallQC pipeline for base calling and quality control. Total reads of 126,780,000 were generated with a Q30 of 97.83% and Q40 of 94.63%. Fastp 0.23.1 (https://github.com/OpenGene/fastp) was used to trim adapters, remove low-quality reads and filter short sequences; second, alignment tools (Bowtie2/BWA) with human genome database were applied to discard reads mapped to human hg38. Kraken2–2.0.7 was used to classify species against database of minikraken2_v2_8GB_201904_UPDATE.

Whole genome sequencing

A total amount of 0.2 μg DNA per sample was used for the DNA library preparations. Briefly, genomic DNA sample was fragmented by Covaris LE220R-plus (Covaris, USA) to a size of 350 bp. Then DNA fragments were endpolished, A-tailed, and ligated with the full-length adaptor for Illumina sequencing, followed by further PCR amplification. The qualified libraries

were pooled and sequenced on Illumina platforms with PE150 strategy. A total of 16.48G clean bases were generated from 9 strains of *H. ducreyi*.

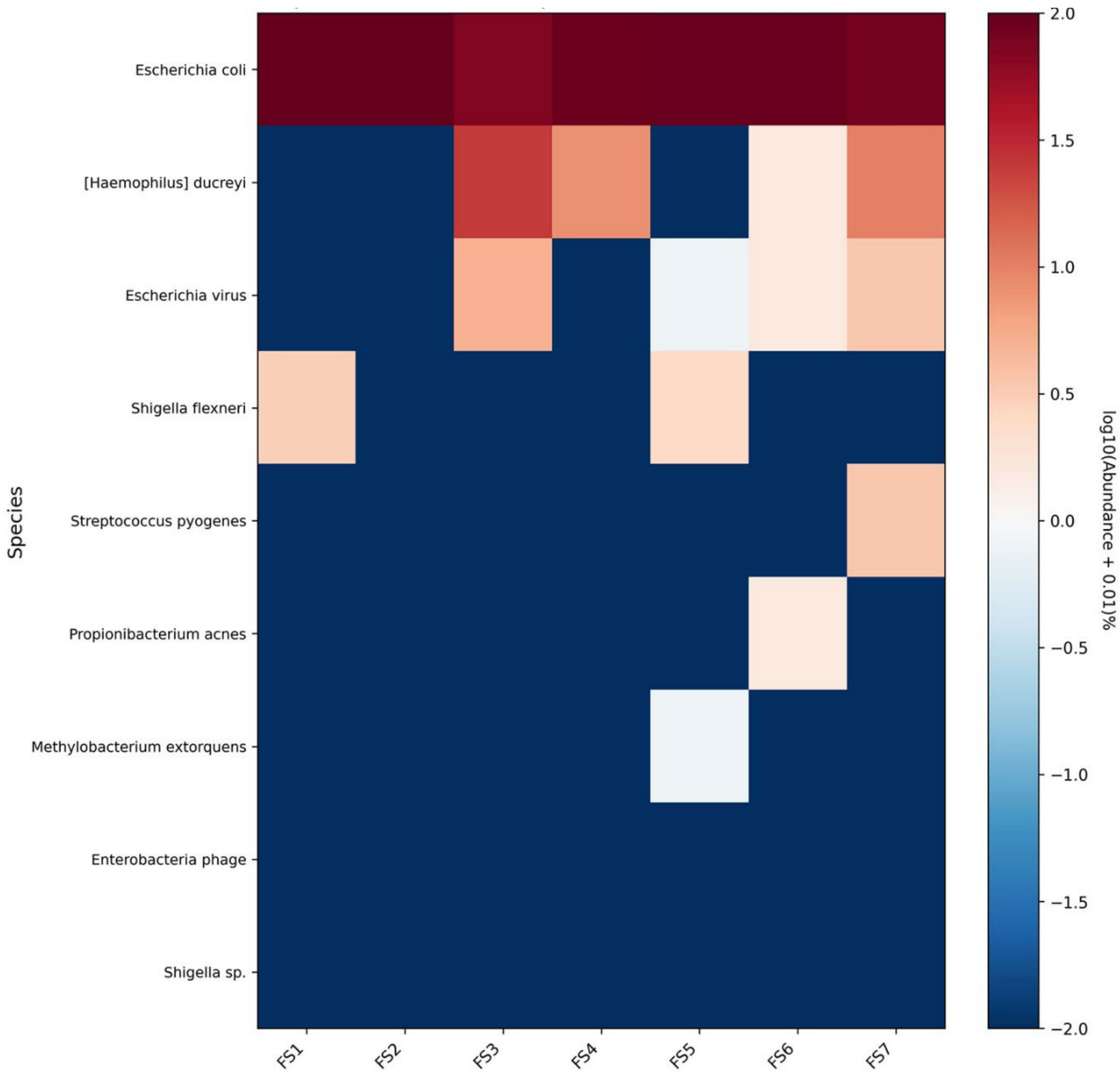

Appendix Figure 1. Metagenomic analysis of the data from the MinION sequencing platform. The heatmap revealed that *H. ducreyi* was detected in 4 of the first 7 skin swab samples. FS, samples collected on 18 November 2025, Sierra Leone.

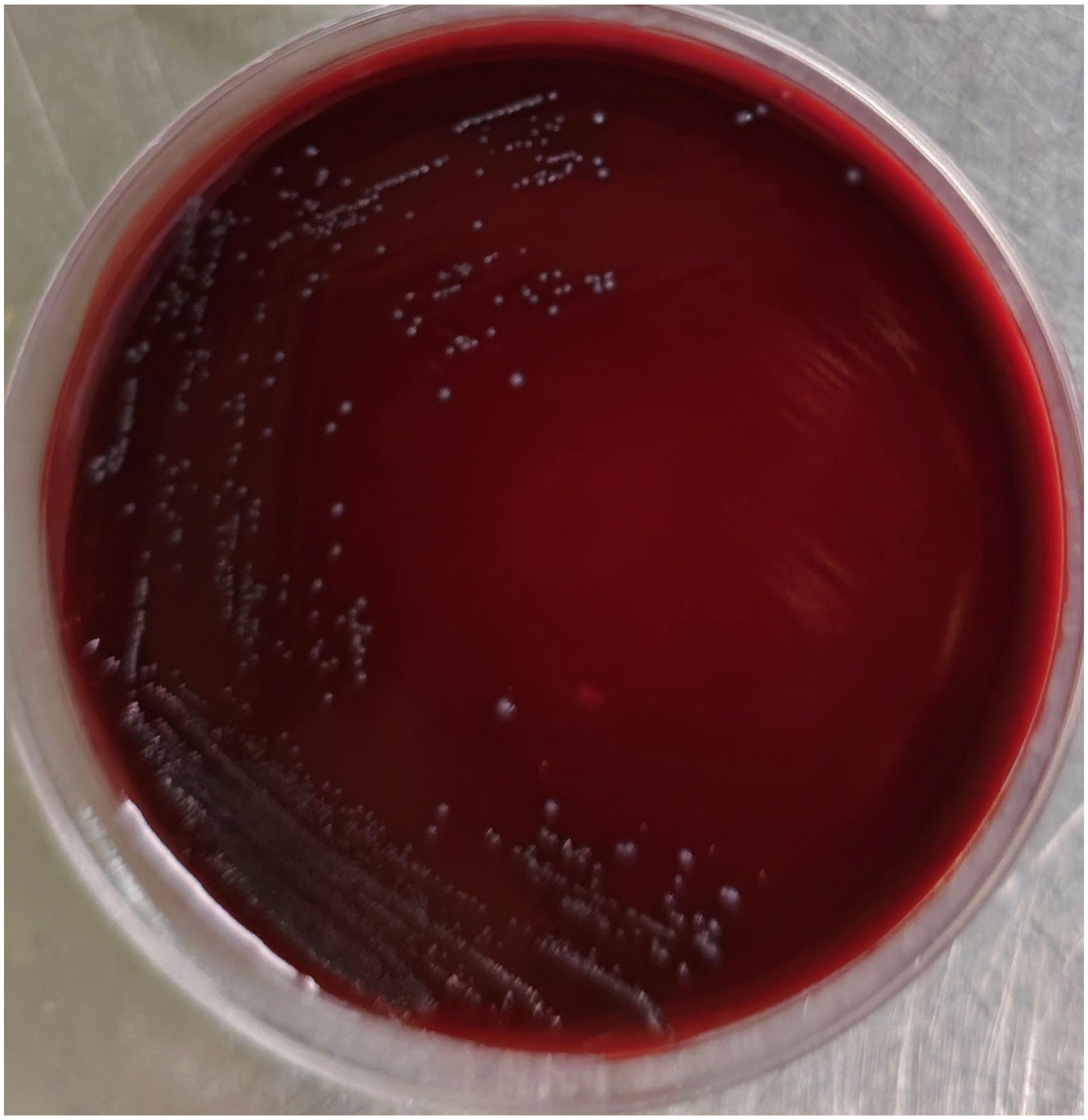

Appendix Figure 2. The bacterial colonies were confirmed as *H. ducreyi* by quantitative PCR after three consecutive subcultures on rabbit blood agar plates, Sierra Leone, December 2025.

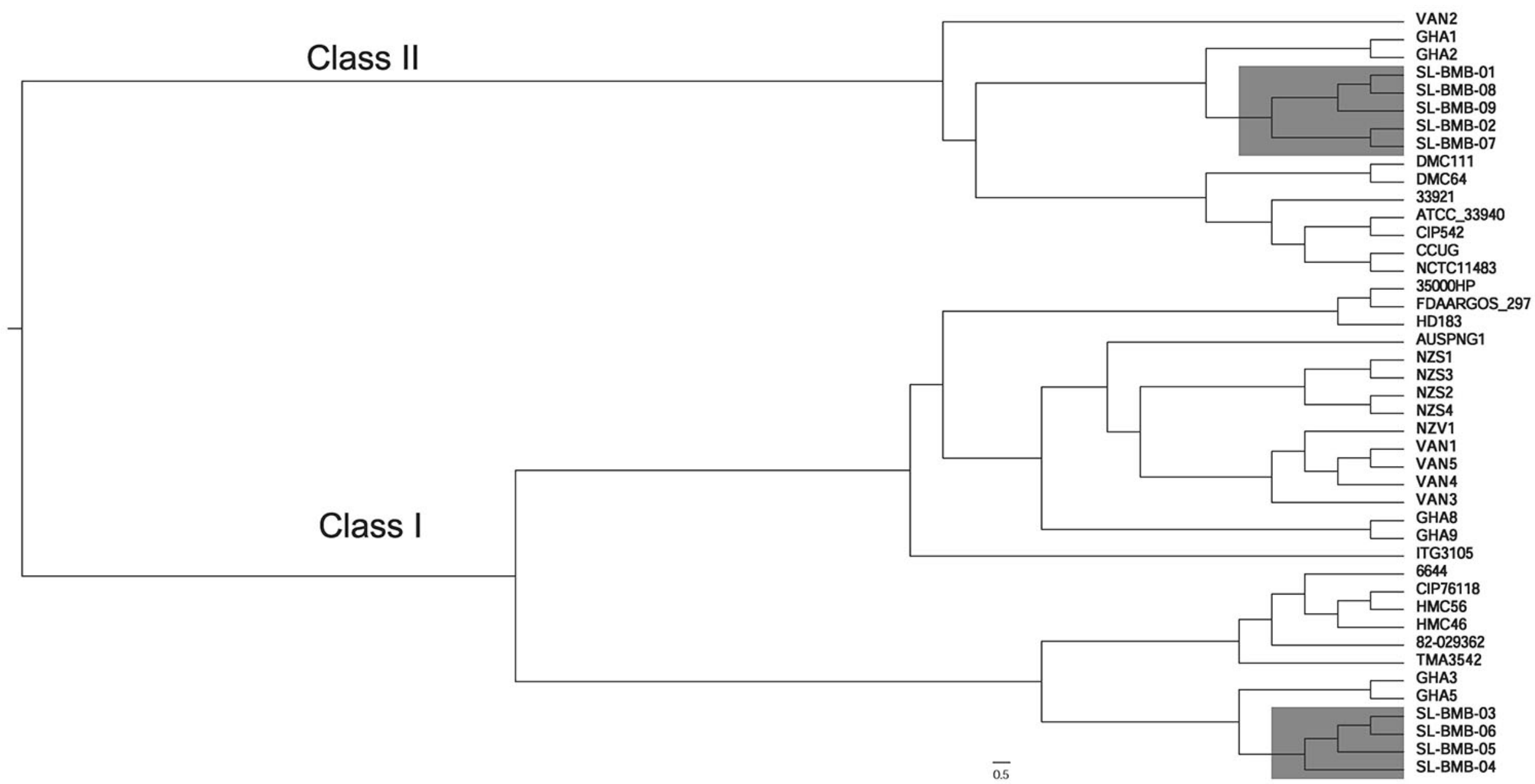

Appendix Figure 3. Phylogenetic analysis on *H. ducreyi*. Whole-genome sequences from nine *H. ducreyi* isolates were combined with 34 publicly available *H. ducreyi* genomes downloaded from GenBank, yielding a total dataset of 43 strains. Core genome SNPs were identified with kSNP4, and a maximum-likelihood phylogenetic tree was built in IQ-TREE v3.3.1 under the GTR+ASC+R10 substitution model with 1000 bootstrap replicates. The resulting tree was visualized and midpoint-rooted in FigTree. Phylogenetic reconstruction revealed that isolates recovered from genital ulcers and cutaneous ulcers distributed across both distinct clades. Notably, all nine Sierra Leonean isolates (shaded) clustered closely with strains recovered from Ghana (another West African nation): four isolates fell within clade I, and the remaining five belonged to clade II, Sierra Leone, December 2025.
